# Novel Mutation in the *PKHD1* Gene Diagnosed Prenatally in a Fetus with Autosomal Recessive Polycystic Kidney Disease

**DOI:** 10.1155/2014/517952

**Published:** 2014-07-13

**Authors:** Pankaj Thakur, Paul Speer, Aleksandar Rajkovic

**Affiliations:** ^1^Division of Medical Genetics, Department of Obstetrics, Gynecology and Reproductive Sciences, Magee-Womens Hospital of University of Pittsburgh Medical Center, 300 Halket Street, Pittsburgh, PA 15213, USA; ^2^Divisions of Ultrasound and Medical Genetics, Department of Obstetrics, Gynecology and Reproductive Sciences, Magee-Womens Hospital of University of Pittsburgh Medical Center, 300 Halket Street, Pittsburgh, PA 15213, USA

## Abstract

We report a 29-year-old gravida 2, para 0100, who presented at 19 weeks and 4 days of gestation for ultrasound to assess fetal anatomy. Routine midtrimester fetal anatomy ultrasound revealed enlarged, hyperechoic fetal kidneys and normal amniotic fluid index. Follow-up ultrasound at 23 weeks and 5 days revealed persistently enlarged, hyperechoic fetal kidneys. Progressive oligohydramnios was not evident until 29 weeks of gestation, with anhydramnios noted by 35 weeks of gestation. Amniocentesis was performed for karyotype and to search for mutations in the *PKHD1* for the presumptive diagnosis of autosomal recessive polycystic kidney disease (ARPKD). In our patient, a maternally inherited, previously reported pathogenic missense mutation in the *PKHD1* gene, c.10444C>T, was identified. A second, previously unreported *de novo* mutation, c.5909-2delA, was also identified. This mutation affects the canonical splice site and is most likely pathogenic. Our case highlights *PKHD1* allelic heterogeneity and the importance of genetic testing in the prenatal setting where many other genetic etiologies can phenocopy ARPKD.

## 1. Introduction

Autosomal recessive polycystic kidney disease (ARPKD; OMIM number 263200) is a single gene, severe hereditary form of polycystic kidney and liver disease caused by mutations in the* PKHD1* gene. It has an estimated incidence of 1 : 40,000 [1] and a carrier frequency of 1 in 100 [2]. ARPKD accounts for approximately 2-3% of the polycystic disease affecting the kidneys, with 75 to 80 percent of cases manifesting in the neonatal period [3].* PKHD1* gene has a complicated transcription profile and undergoes complex alternative splicing that generates multiple variants of the fibrocystin/polyductin (FC/PD) protein [4, 5]. Given that mutations are spread throughout the* PKHD1* gene and not clustered at a region, the clinical outcome may be difficult to predict based on the molecular genetic data alone. Because most of the* PKHD1* mutations are unique to individual families, genotype-phenotype correlation is not well established. Approximately 30 to 50 percent of newborns die shortly after birth, secondary to pulmonary hypoplasia [6]. The perinatal form of ARPKD usually presents at birth with bilateral enlarged cystic kidneys leading to severe renal failure. Antenatal ultrasound has remarkably improved our understanding of the natural history of the disease. Many cases present in the second trimester of pregnancy with enlarged, hyperechoic kidneys, accompanied by oligohydramnios or even anhydramnios. Progressive oligohydramnios may lead to positional limb deformities, flattened facial profile with broad nasal bridge, recessed chin, pseudoepicanthic folds, and pulmonary hypoplasia, also known as Potter sequence in severely affected fetuses. However, mutations in HNF1B or genes that typically cause other ciliopathies, such as nephronophthisis, Bardet Biedl, Joubert syndrome, and related disorders, can mimic ARPKD. Prenatal ultrasound based renal phenotyping cannot accurately distinguish between different genetic etiologies and genetic testing is therefore critical to make accurate diagnosis that can affect obstetric and neonatal management.

We present a case of ARPKD, initially diagnosed at 19 weeks and 4 days of gestation by ultrasound. The newborn died of pulmonary hypoplasia soon after delivery, likely secondary to oligohydramnios first identified at approximately 29 weeks of gestation.* PKHD1* mutational analysis revealed a maternally inherited c.10444C>T recurrent missense mutation, previously reported to be associated with ARPKD [7]. An additional, previously unreported,* de novo* mutation, c.5909-2delA, was also present. Our case report highlights the importance of routine antenatal ultrasound and molecular genetic testing in clinically diagnosing ARPKD to establish appropriate prenatal management.

## 2. Material and Methods

Chromosome analysis was performed on metaphase spreads prepared from the cultured amniotic fluid specimen using standard laboratory protocols at the Pittsburgh Cytogenetics Laboratory. Fifteen cells were analyzed at the 500 G-band level of resolution to determine karyotype. Targeted mutational analysis for the* PKHD1* gene was performed by PCR based exon amplification and direct bidirectional sequencing of both strands of all 67* PKHD1* exons at the Medical Genomics Laboratory, University of Alabama at Birmingham.

## 3. Clinical Report

The proband is a 29-year-old gravida 2, para 0100, who presented at 19 weeks and 4 days of gestation for routine ultrasound to assess fetal anatomy. She and her husband were healthy and nonconsanguineous and denied exposure to any teratogens. A three-generation pedigree did not reveal significant history of renal disease. Her obstetric history was significant for a previous pregnancy with loss of a fetus with an omphalocele at 18 weeks of gestational age and normal chromosomal microarray.

**Figure 1:**
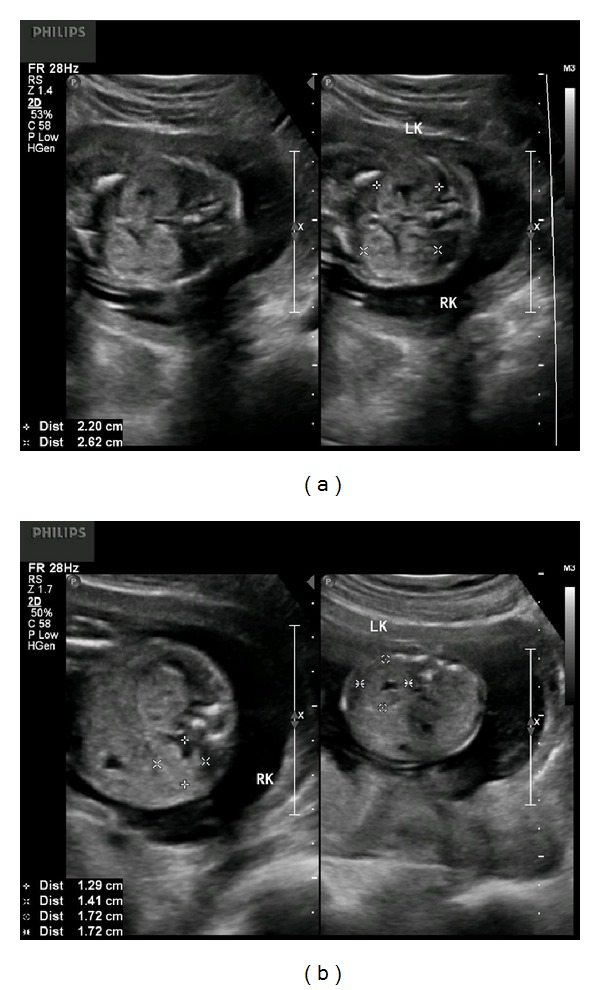
(a) Coronal images of the fetal kidneys at 19 weeks and 4 days of gestation. The kidneys are enlarged and echogenic. (b) Transverse images of the fetal kidneys at 19 weeks and 4 days of gestation. The amniotic fluid level was normal for gestational age.

The current pregnancy began uneventfully until routine midtrimester fetal anatomy ultrasound revealed enlarged, hyperechoic kidneys. The renal volume bilaterally was greater than the 90th percentile for gestational age (Figures 1(a) and 1(b)).

The remaining fetal biometry and anatomy survey was unremarkable. The ultrasound findings were suspicious for ARPKD. The amniotic fluid level of 145 mm, however, was within normal limits for gestational age. The follow-up ultrasound at 23 weeks and 5 days revealed persistently enlarged and hyperechoic fetal kidneys with normal amniotic fluid index measuring 115 mm. The patient was counseled regarding the suspicion for ARPKD and given options for genetic testing. She elected to undergo amniocentesis for fetal karyotype and mutation detection in the* PKHD1* gene for the presumptive diagnosis of ARPKD at 26 weeks and 4 days of gestational age. She elected to continue the pregnancy and was followed through the Center for Advanced Fetal Diagnostics at Magee-Womens Hospital. Karyotype was normal 46, XX. Mutation analysis of the* PKHD1* gene revealed a maternally inherited and previously reported c.10444C>T (p.Arg3482Cys) missense mutation and a novel, previously unreported* de novo* splice site mutation, c.5909-2delA.

**Figure 2:**
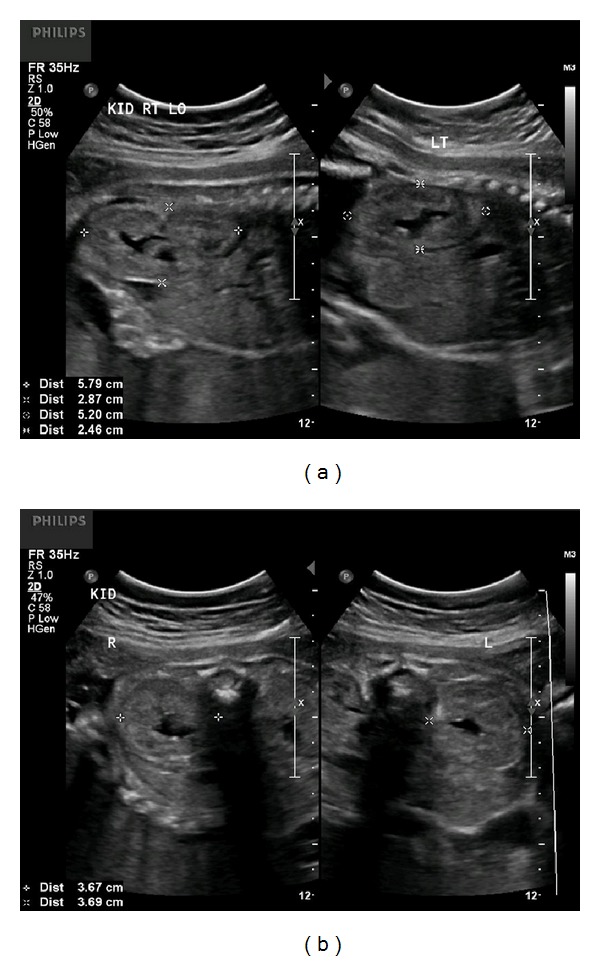
(a) Longitudinal images of the fetal kidneys at 29 weeks and 1 day of gestation. The kidneys continue to be enlarged and echogenic in appearance. Oligohydramnios was noted at the time of this ultrasound. (b) Transverse images of the fetal kidneys at 29 weeks and 1 day of gestation.

**Figure 3:**
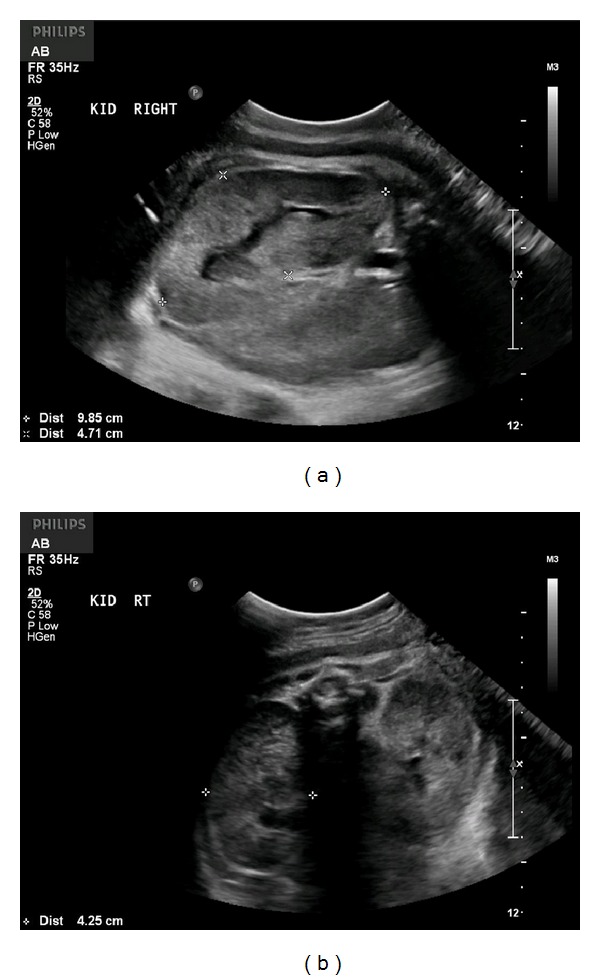
(a) Longitudinal image of the fetal right kidney at 35 weeks and 1 day of gestation. Notice the significantly enlarged renal size. (b) Transverse image of the fetal kidneys at 35 weeks and 1 day. Notice how the kidneys essentially occupy the entire fetal abdomen. Anhydramnios was noted at this gestational age.

Follow-up ultrasound arranged at 2-week intervals revealed persistent, enlarged, and hyperechoic kidneys, with oligohydramnios (AFI 46 mm), first identified at 29 weeks (Figures 2(a) and 2(b)), followed by anhydramnios identified at 35 weeks of gestation (Figures 3(a) and 3(b)). Fetal growth was noted to be appropriate throughout gestation (Table 1).

At 29 weeks of gestation, the fetal abdominal circumference was noted to be at the 90th percentile for gestational age, and by 32 weeks, the fetal abdominal circumference measured greater than the 97th percentile, likely due to the enlarged kidneys. A 3366-gram female neonate with APGAR scores of 4 and 8 at 1 and 5 minutes, respectively, was delivered vaginally at 35 weeks and 5 days following induction of labor. The newborn required intubation for respiratory distress at two minutes after delivery. The neonate continued to have persistent hypoxemia and hypercarbia despite aggressive mechanical ventilation. Chest X-rays done after birth revealed low lung volumes with bilateral pneumothorax. The family elected for palliative care and the newborn was extubated approximately three hours following delivery and died soon thereafter. An autopsy was offered, which the family declined.

## 4. Discussion

ARPKD is caused by mutations in the* PKHD1* gene, which maps to chromosome 6p21.1-p12.* PKHD1* is amongst the largest genes in humans with 66 coding exons [5] and encodes a 4074-amino acid protein named fibrocystin/polyductin (FC/PD). FC/PD is expressed in kidneys, adrenal glands, liver, and pancreas. FC/PD is a single transmembrane domain protein with a short intracellular carboxy terminus and a large extracellular amino terminus [5]. FC/PD has been shown to be localized to the primary cilia with concentrations in the basal body [8, 9]. Mutations within FC/PD cause abnormal epithelial differentiation, followed by increased apoptosis and fluid secretion that ultimately lead to clinically detectable cyst formation [6, 10]. Due to high incidence of private mutations, the clinical outcome is not predictable based on the molecular genetic data. The reported mutation detection rate is about 80 percent for the entire clinical spectrum of ARPKD patients [1, 2]. The rest of the mutations are likely in the regulatory regions that are not captured by current sequencing strategies.

In our case, mutational analysis of the* PKHD1* gene revealed a maternally inherited c.10444C>T (p.Arg3482Cys) missense mutation in the* PKHD1* gene, reported several times in the literature and, in all cases, as pathogenic. However, a second previously unreported* de novo* c.5909-2delA splicing mutation was also found and inherited paternally. The c.5909-2delA variant is not present in the exome variant server, dbSNP135, or HGMD. The c.5909-2delA mutation disrupts the exon 37 splice acceptor canonical site. The exon 37 splice acceptor site is conserved in frogs and other vertebrates. The next predicted splice acceptor site (http://www.fruitfly.org/seq_tools/splice.html) is 25-nucleotide downstream and leads to premature stop codon insertion. The mutation is therefore likely to produce truncated protein product. Carrying two truncating mutations is usually lethal, whereas the presence of at least one missense mutation is compatible with life due to residual functioning fibrocystin protein [6]. In our case, one mutation is previously reported missense while the other is predicted to be a novel splice mutation. Homozygous p.Arg3482Cys missense mutation was previously reported to be associated with perinatal lethal phenotype in two consanguineous Israel-Arab families [7]. It is therefore not surprising that missense p.Arg3482Cys and splicing c.5909-2delA mutations led to perinatal mortality.

The fetus in our case was suspected to have ARPKD on routine fetal anatomy ultrasound. However, it is important to emphasize that other genetic etiologies can phenocopy ARPKD renal findings. These disorders include HNF1B and the diverse group of ciliopathies, such as nephronophthisis, Bardet Biedl, and Joubert syndrome. The antenatal imaging modality of choice to monitor for progression of disease is serial ultrasound assessment of the fetus. MRI has also been used for better phenotyping of renal anatomy. MRI will show enlarged kidneys with hyperintense T2-weighted signals in ARPKD cases [11]. MRI may be more sensitive than ultrasound in differentiating between the cysts, normal parenchyma, and fibrosis, although higher cost limits its use in prenatal imaging. In the present case, follow-up ultrasound at 2-week intervals revealed progressively declining amniotic fluid, with oligohydramnios beginning at 29 weeks and anhydramnios by 35 weeks of gestation.

Thirty percent of the newborns diagnosed with ARPKD die during the neonatal period due to pulmonary hypoplasia. The use of a ventilator has been suggested as a prognostic marker indicative of lower survival [2, 3]. After the baby is born, resuscitation is the key, as well as careful physical examination to rule out other structural birth defects that may point to etiologies other than ARPKD. Our patient had severe respiratory distress at the time of delivery. Following intubation, the newborn required high ventilator settings but had persistent hypoxemia and hypercarbia and subsequently developed bilateral pneumothoraces, all related to underlying pulmonary hypoplasia. Survival past the neonatal period has a more favorable outcome, with 10-year survival rate of approximately 82 percent [12]

Given the poor prognosis and severity of ARPKD, the option of prenatal testing in pregnancy and preimplantation genetic diagnosis (PGD) in any subsequent pregnancies must be discussed in couples known to be carriers of ARPKD mutations. PGD using a single embryo genome amplification with multiple displacement amplification (MDA) and haplotype analysis with novel short tandem repeat (STR) markers from the* PKHD1* gene have been successfully done in the past for known mutations [13, 14]. ARPKD has a variable clinical spectrum, but in a given family, patients usually display comparable phenotypes. The affected newborn in our case was the first affected member of the family. With more cases of ARPKD being diagnosed prenatally, there is ongoing research for therapies to halt disease progression in the future, but currently none is available.

Our case emphasizes the importance of prenatal diagnosis of renal abnormalities by routine fetal anatomic ultrasound and genetic testing for ARPKD. Because other conditions can mimic ARPKD, the use of genetic panels will likely become the preferred option for genetic testing. Our study presents a detailed change in the fetal biometry over the prenatal course of the perinatal ARPKD with a novel splice mutation. In our case, oligohydramnios was not present until 29 weeks of gestational age, followed by anhydramnios. The earlier oligohydramnios or anhydramnios is diagnosed, the higher likelihood of a poor neonatal outcome is.

## Figures and Tables

**Table 1:** Pregnancy and fetal growth data.

Gestational age (based on a LMP)	Biometric measurements	Composite gestational age	Amniotic fluid index	Fetal kidney measurement	Comments
7.3 weeks	Gestational sac: 2.6 cm CRL: 1.1 cm	NA	NA	NA	Crown rump length is consistent with the patient's gestational age by LMP

19.4 weeks	BPD: 4.9 cm HC: 17.8 cm AC: 16.1 cm FL: 3.4 cm Tibia: 2.9 cm Humerus: 3.2 cm Ulna: 3.0 cm Radius: 2.7 cm	20.7 weeks	14.5 cm (normal)	Right kidney: 2.3 × 1.3 × 1.4 cm (>90th percentile) Left kidney: 2.2 × 1.7 × 1.7 cm (>97th percentile)	Bilateral enlarged echogenic fetal kidneys

23.5 weeks	NA	NA	11.5 cm (normal)	Right kidney: 3.9 × 2.4 × 2.4 cm (>97th percentile) Left kidney: 3.7 × 2.1 × 2.0 cm (>97th percentile)	(1) Bilateral enlarged and echogenic fetal kidneys (2) No sonographic evidence of dilation of the renal pelvis or hydro ureter

29.1 weeks	AC: 27.3 cm FL: 6.0 cm Fetal weight: 1788 gms (86th percentile)	31.3 weeks	4.6 cm (oligohydramnios)	Right kidney: 5.8 × 2.9 × 3.7 cm Left kidney: 5.7 × 2.8 × 3.7 cm	(1) Bilateral enlarged and echogenic fetal kidneys (2) Oligohydramnios

35.1 weeks	NA	NA	0 cm (anhydramnios)	NA	(1) Bilateral enlarged and echogenic fetal kidneys essentially filling the entire fetal abdomen (2) Anhydramnios

^1^AC: abdominal circumference.

^2^CRL: crown rump length.

^3^BPD: biparietal diameter.

^4^FL: femur length.

^5^HC: head circumference.
